# Biophysical Manipulation of the Extracellular Environment by *Eurotium halophilicum*

**DOI:** 10.3390/pathogens11121462

**Published:** 2022-12-02

**Authors:** Anna Micheluz, Flavia Pinzari, Edgard G. Rivera-Valentín, Sabrina Manente, John E. Hallsworth

**Affiliations:** 1 Conservation Science Department, Deutsches Museum, Museumsinsel 1, 80538 Munich, Germany; 2 Institute for Biological Systems, Council of National Research of Italy, Area della Ricerca di Roma 1, Via Salaria Km 29,300, 00015 Monterotondo, Italy; 3 Life Sciences Department, Natural History Museum, Cromwell Road, London SW7 5BD, UK; 4 Johns Hopkins University Applied Physics Laboratory, Laurel, MD 20723, USA; 5 Department of Molecular Sciences and Nanosystems, Scientific Campus, Ca’ Foscari University of Venice, Via Torino, 30170 Venice, Italy; 6 Institute for Global Food Security, School of Biological Sciences, Queen’s University Belfast, 19 Chlorine Gardens, Belfast BT9 5DL, UK

**Keywords:** astrobiology, extracellular polymeric substances (EPS), *Eurotium halophilicum*, global climate change, library-book contamination, model xerophile species, the built environment, water activity

## Abstract

*Eurotium halophilicum* is psychrotolerant, halophilic, and one of the most-extreme xerophiles in Earth’s biosphere. We already know that this ascomycete grows close to 0 °C, at high NaCl, and—under some conditions—down to 0.651 water-activity. However, there is a paucity of information about how it achieves this extreme stress tolerance given the dynamic water regimes of the surface habitats on which it commonly occurs. Here, against the backdrop of global climate change, we investigated the biophysical interactions of *E. halophilicum* with its extracellular environment using samples taken from the surfaces of library books. The specific aims were to examine its morphology and extracellular environment (using scanning electron microscopy for visualisation and energy-dispersive X-ray spectrometry to identify chemical elements) and investigate interactions with water, ions, and minerals (including analyses of temperature and relative humidity conditions and determinations of salt deliquescence and water activity of extracellular brine). We observed crystals identified as eugsterite (Na_4_Ca(SO_4_)_3_·2H_2_O) and mirabilite (Na_2_SO_4_·10H_2_O) embedded within extracellular polymeric substances and provide evidence that *E. halophilicum* uses salt deliquescence to maintain conditions consistent with its water-activity window for growth. In addition, it utilizes a covering of hair-like microfilaments that likely absorb water and maintain a layer of humid air adjacent to the hyphae. We believe that, along with compatible solutes used for osmotic adjustment, these adaptations allow the fungus to maintain hydration in both space and time. We discuss these findings in relation to the conservation of books and other artifacts within the built environment, spoilage of foods and feeds, the ecology of *E. halophilicum* in natural habitats, and the current episode of climate change.

## 1. Introduction

Global climate change is causing perturbations and reducing the predictability of planetary-scale meteorology and local weather patterns. In addition, the amount of water retained by Earth’s warming atmosphere is increasing, thus affecting the hydrological cycle [1,2]. The microbial biosphere is undergoing large-scale changes that are impacting the microbial communities of the natural environment and those within the built environment [3], some of which are predictable and some of which are not. For some microorganisms, their habitat buffers them against the extremes of global warming, including those in the deep ocean where there are no significant changes in temperature, microbe-water relations, or ultra-violet radiation. By contrast, fungal xerophiles and other microbes that live on exposed surfaces (at the planetary surface) can be subject to major changes in the spatial and temporal availability of water. The current study focuses on a biophysically unique fungal xerophile, *Eurotium halophilicum*, with a robust stress biology that is characterised by resilience to multiple stresses. We suspect that the ability of this fungus to cope with changing conditions will determine its persistence within both natural ecosystems and anthropogenic environments.

*Eurotium halophilicum* C.M. Chr., Papav. & C.R. Benj. is an ascomycete that was first described by C.M. Christensen et al. in 1954 and has been isolated from foodstuffs with low water-activity values (such as stored wheat [4] and cardamom seeds [5]), in atmospheric particulate matter [6], in house dust [7] in salted ham [8], in Fuzhuan fermented tea [9], and within the built environment [10,11,12,13,14,15]. It is obligately xerophilic [5,16,17] so is not readily cultured on standard (high water-activity) nutrient media. Therefore, it has been mistakenly thought of as an environmentally rare microorganism.

**Figure 1:**
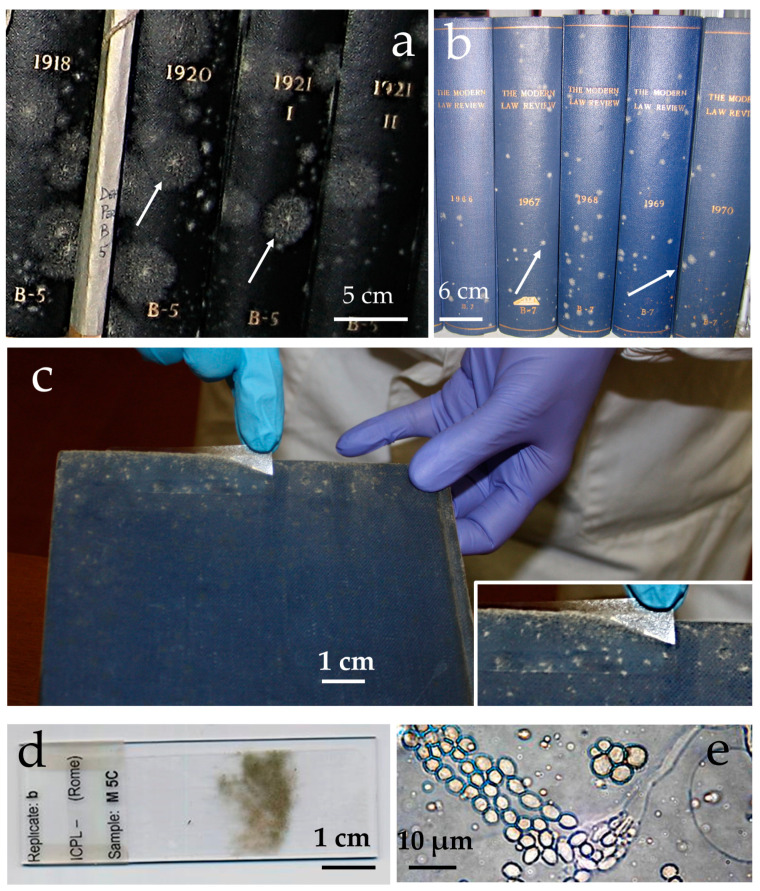
Colonies of *E. halophilicum* on the external surfaces of books in Venice, Italy (**a**,**b**); arrows show examples of colonies. The sampling of *E. halophilicum,* using adhesive tape (**c**); adhesive tape containing the fungal mycelium attached to a glass slide (**d**); and *E. halophilicum* conidiophore and conidia sampled with adhesive tape and viewed under a light microscope (**e**).

*Eurotium halophilicum* has been found worldwide in indoor environments such as archives, libraries and museums, such as the surfaces of books (Figure 1), wood, leather, and paintings [13,18,19]. Direct observation of indoor-grown mycelium under the light microscope has often shown the presence of conidiophores and conidia belonging to the anamorphic state of *E. halophilicum*, i.e., *Aspergillus halophilicus* [7], but also mature ascocarps of the perfect form [12]. *Eurotium halophilicum* is extremely cold tolerant (under some conditions growing close to 0 °C) [20] and is amongst the 10 or so most-xerophilic microbes known [21,22]. It has been observed germinating and producing hyphae at 0.651 water activity on media supplemented with glycerol, NaCl and sucrose [17]. Water activity, the effective mole fraction of water, represents a potent biophysical constraint for life on Earth, so this ability of *E. halophilicum* is remarkable [17,21].

*Eurotium halophilicum* hyphae are generally covered with hair-like microfilaments, especially when growing at low water activity, that are several nanometres in diameter. The purpose of these structures, and the physiological and behavioural adaptations of *E. halophilicum* to saline conditions and low water activity, remain enigmatic. Here, we carried out studies to determine biophysical aspects of *E. halophilicum* ecology at low water activity. The specific aims were to retrieve *E. halophilicum* from the surfaces of library-stored books, examine the morphology of this *E. halophilicum* that had developed at low water activity, characterise its extracellular environment including any extracellular polymeric substances and salt crystals present, and seek evidence the water-relations strategy that *E. halophilicum* uses to facilitate its extreme xerophilicity.

## 2. Results

**Figure 2:**
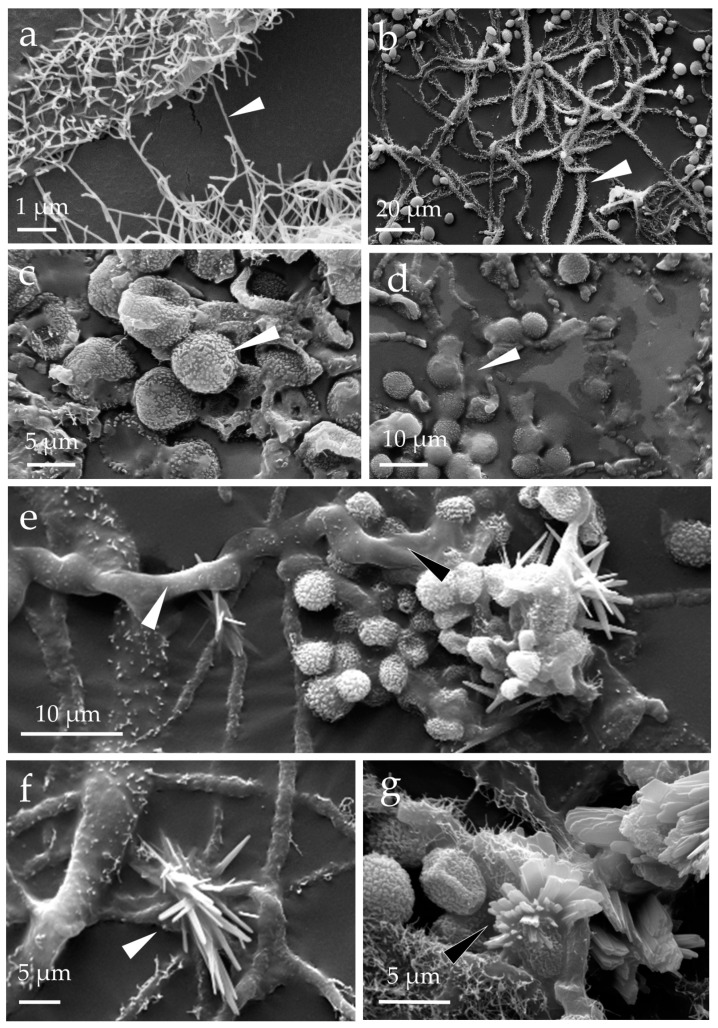
*Eurotium halophilicum* mycelium taken from books stored in Venice, Italy (that had been kept at a mean temperature of 22.7 ± 0.5 °C) and observed with a scanning electron microscope (SEM EVO50 Zeiss, Oxford Instruments, Abingdon, UK) in high vacuum mode, after metallisation with gold: (**a**,**b**) detail of the hyphae characterised by a covering of hair-like microfilaments (see the arrows); (**c**) echinulate conidia of *Aspergillus halophilicus*, the anamorph of *E. halophilicum* (indicated by the arrows); (**d**,**e**) presence of extracellular polymeric substances around the fungal structures (see the arrows); and (**f**,**g**) crystals formed around hyphae and clusters of conidia (see the arrows).

The *E. halophilicum* hyphae retrieved from the library books (Figure 1) were covered with fine microfilaments that are acellular (Figure 2a,b). The samples were uniformly metallised upon the addition of gold during sample preparation, and the textured (echinulate) surfaces of the conidia were readily observed (Figure 2c). The images also revealed the presence of a gelatinous matrix enveloping some of the fungal structures, apparently made up of extracellular polymeric substances (Figure 2d,e). The mycelium not pre-treated prior to coating with gold, indicating that this gelatinous matrix was not an artifact created by sample preparation. The mass of extracellular polymeric substances was often associated with crystals that are the shape of needles and flat raphides (Figure 2f,g).

**Figure 3:**
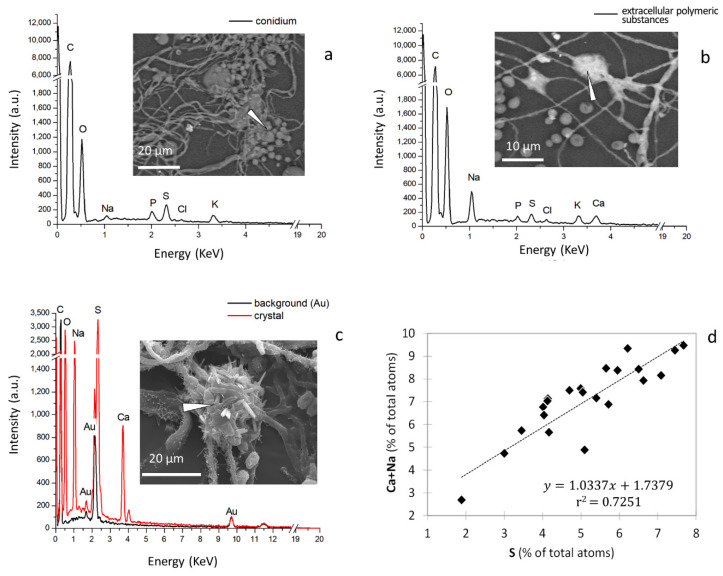
*Eurotium halophilicum* structures (taken from books that had been kept at a mean temperature of 22.7 °C) under SEM (EVO50 Zeiss, Oxford Instruments, Abingdon, UK): images (**a**,**b**) were obtained in variable pressure mode, without any pre-treatment of the sample, and image (**c**) was obtained in high vacuum mode, after metallisation with gold. The energy-dispersive X-ray spectra were obtained with an Oxford INCA system and show the elemental composition of objects visualised and selected within the SEM images. Arrows in (**a**–**c**) indicate the areas that were analysed. The counts (*y* axis) are arbitrary units (a.u.) of signal intensity; image (**d**) is a scatter plot of the concentrations of S atoms versus those of Na + Ca atoms as a percentage of total atoms present in the glauberite crystals. The strong correlation indicates a constant ratio between the elements, which suggests the empirical formula of the mineral glauberite: Na_2_Ca(SO_4_)_2_. The data analysed in plot (**d**) were derived from samples taken in all five libraries (for the raw data, see the link provided in the Data Availability Statement).

The fungal structures (that are not metallised) observed in variable pressure mode and using a backscattered electrons detector appeared lighter than the background (Figure 3a,b). This background consists of the sticky surface of the adhesive tape that is made of the carbon-based polymer polypropylene and a water-based acrylic glue contains only H, C and O atoms. The contrast between the mycelium and the background indicates that the mycelium contains elements with an atomic number higher than that of carbon. Energy-dispersive X-ray spectroscopy measurements, performed by pointing the detector at the different structures visible in the scanning electron microscope (SEM) images, showed that conidia and extracellular polymeric substances have different compositions (Figure 3).

**Figure 4:**
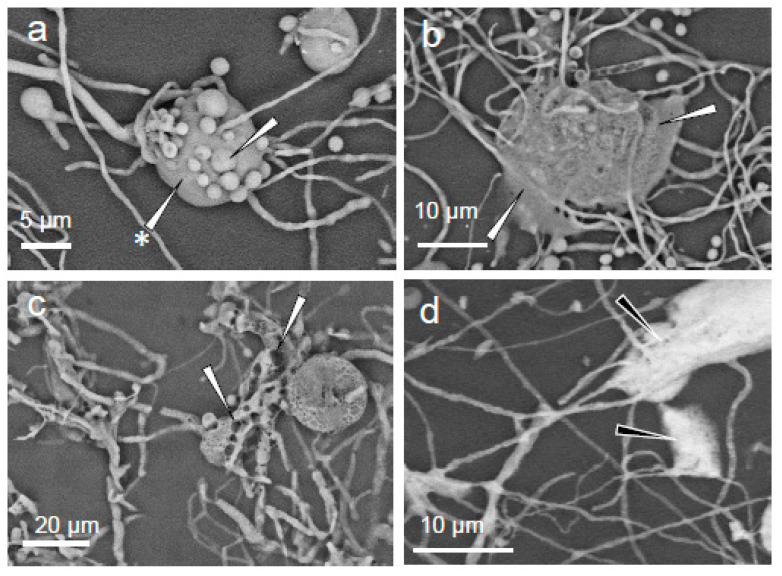
*Eurotium halophilicum* structures (sampled directly from books in Rome Library 1, Italy (that had been kept at a mean temperature of 17 ± 1.6 °C) under SEM (EVO50 Zeiss, Oxford Instruments, Abingdon, UK) using a backscattered electrons detector; the images were obtained in variable pressure mode without any pre-treatment of the sample: (**a**) cluster of conidia (arrow) embedded in extracellular polymeric substances (arrow with asterisk); (**b**) extracellular polymeric substances after the application of pressure (see arrows); (**c**) foamy appearance of the extracellular polymeric substances after prolonged exposure to the electron beam (see arrows); and (**d**) regions where the extracellular polymeric substances appear rich in elements with a relatively high atomic number (indicated by the white areas towards; see black arrows).

The extracellular polymeric substances are evident in several samples as gelatinous masses, sometimes appearing as globules surrounding clusters of hyphae and conidia (Figure 4a). If the adhesive tape was gently pressed with a cotton-tipped swab when sampling, these globules became flattened (Figure 4b). Prolonged exposure to the heat of the electron beam during energy-dispersive X-ray spectroscopy measurements caused the boiling of the extracellular polymeric substances, which then retained a new (foam-like) consistency (Figure 4c). In some samples, the extracellular polymeric substances appeared light in tone (see Figure 4d) indicating that chemical elements with a high atomic number (in this case, salts) are at high concentrations.

Further energy-dispersive X-ray spectroscopy measurements were carried out on conidia, extracellular polymeric substances, and background (the adhesive tape) using samples obtained from different books kept in libraries in Italian cities (see *Materials and Methods*). This enabled us to obtain a dataset of chemical compositions for indoor populations of *E. halophilicum* (see the link provided in the Data Availability Statement). These data were recorded as the weight per cent of each chemical element (i.e., % of total elements, based on atomic mass) by normalising the overall elemental content of each observation to 100. The background adhesive tape was analysed to compare with the data obtained from analyses of fungal structures, extracellular polymeric substances, and salt crystals to ensure that, in each case, the beam only landed on the target structure (and there was no background inadvertently included in the area being analysed). We expected no elements in the background apart from carbon and oxygen (hydrogen is not measured), so the detection of small concentrations of other elements indicates a signal scattering from areas of the sample.

**Figure 5:**
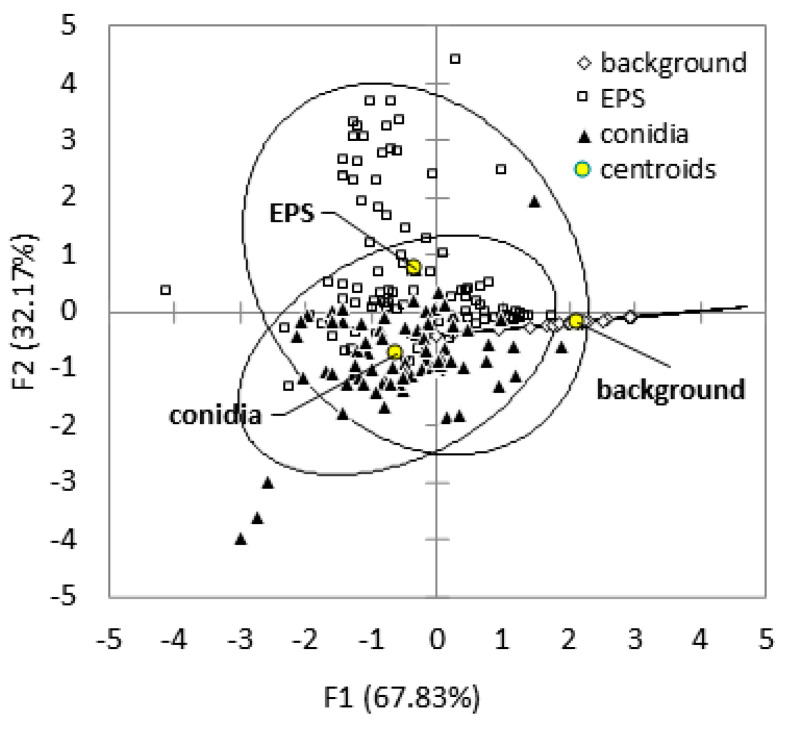
Discriminant analysis plot showing the clustering and classification of conidia, extracellular polymeric substances (EPS), and background based on their elemental composition as determined by energy-dispersive X-ray spectroscopy. The group centroid is the mean value of the discriminant score for a given category. The confidence ellipses (confidence interval 95%) are shown using solid black outlines for each category; these appear as oval shapes for EPS and conidia, and a linear shape for the background. The data analysed were derived from samples taken in all five libraries (for the raw data, see the link provided in the Data Availability Statement).

The elemental compositions of the conidia, the extracellular polymeric substances, and the background were compared by ANOVA followed by a post hoc test for *p* < 0.05 (Table 1). The conidia (Figure 3a) contained mainly phosphorus (P) and potassium (K) while the extracellular polymeric substances (Figure 3b) are rich in sodium (Na), sulphur (S), and chlorine (Cl). The salt crystals contained sodium (Na), sulphur (S), and calcium (Ca) (Figure 3c). Conidia contain significantly more P and K than the extracellular polymeric substances (*p* < 0.001) whereas the latter contained more Na, S, and Ca than the former. The element Cl was present in both conidia and the extracellular polymeric substances at comparable concentrations. The analysis of the background (adhesive tape) indicated traces of S but this is likely due to scattering of electrons from fungal structures. The chemical elements were used as variables in a principal component analysis (Supplementary Figure S2) to estimate the correlations between them. A discriminant analysis was then run using the factor scores and the first three new orthogonal components resulting from the principal component analysis. In total, the new components (F1, F2 and F3) accounted for 84.2% of the variance in the energy-dispersive X-ray spectroscopy dataset (Table 2). The results of the discriminant analysis are shown in Figure 5, with the conidia classified as 84.21% distinct in their identity (considered ‘correctly classified’), and the extracellular polymeric substances classified as 72.04% (Table 3).

The Pearson correlation between the chemical elements (Table 4) revealed a strong reciprocal correlation between Na, S, and Ca (Pearson’s coefficient R = 0.82 between Na and S, lower correlation values between Ca and S, or Na, but still statistically significant for *p* < 0.05). The correlation between P and K was close to 1 (R = 0.97).

The X-ray powder diffraction analysis conducted on the crystals associated with the *E. halophilicum* mycelium included peaks characteristic of eugsterite and mirabilite, but none for glauberite (Supplementary Figure S3, Supplementary Tables S1 and S2). The radiation beam may be inconsistent with the size of the crystals, which were only a few tens of microns in diameter (Figure 2) so gave a weak signal and the presence of organic material in the vicinity of the crystals (hyphae, conidia, extracellular polymeric substances) may have interfered with the diffraction signal. When X-rays pass through the crystal structure, their diffraction angle is diagnostic for each mineral type. However, if a sample is heterogenous, the X-rays have numerous wavelengths so different forms of interference may occur, and the diffracted beams can cancel each other out [23]. From our sample, the signals generated some characteristic peaks in the initial part of the acquisition spectrum but not in its final part.

## 3. Discussion

The current study revealed that *E. halophilicum* growing on the surfaces of book surfaces is able to produce healthy-looking colonies that consist of hyphae, conidia, and extracellular polymeric substances. Furthermore, we found these colonies to be intimately associated with salt crystals despite the fact that the fungus is living on surfaces that look dry to the human eye rather than in visible body of brine water. This remarkable ecology raises intriguing questions about how *E. halophilicum* relates to its apparently stressful biophysical environment and yet manages to flourish. Of particular import seems to be the relationship between the fungus, ions and salts, vapour-phase water, and waters-of-deliquescence that are created when salt crystals absorb water vapour and turn into a thin film of brine (not visible to the naked eye; see Movie S1 of [22]).

We observed sodium sulphate crystals in the vicinity of *E. halophilicum* (Figure 2e–g; Supplementary Figure S2a,b). Given their small size, the minerals could not be definitively identified but the stoichiometry of the elements suggested that in many samples the crystals observed were eugsterite and mirabilite (Figure 3d, Supplementary Figure S3). The formation of sodium sulphate minerals also occurs in some natural environments and has been reported, for example, on the rock surfaces within caves systems [24]. They are minerals that oscillate between deliquescence (to form brines) and recrystallisation from brines, so are known as metastable; their state depends on the prevailing temperature and relative humidity [25].

The X-ray diffraction analysis identified 14 peaks out of 93 for eugsterite and six out of 32 for mirabilite (Supplementary Figure S3). Eugsterite is a monoclinic sodium-calcium-sulphate-hydrate mineral with formula Na_4_Ca(SO_4_)_3_·2H_2_O that forms clusters of thin fibres. It was first discovered in saline soils in Kenya and Turkey, in salt efflorescences on bricks, and occurs in association with halite, gypsum and glauberite [26]. In contrast, mirabilite is a hydrated sodium sulphate (Na_2_SO_4_-10H_2_O) that is a monoclinic mineral that precipitates from sodium-sulphate-saturated waters including Great Salt Lake (Utah, USA) and some other brine lakes (and can also be associated with other minerals such as include gypsum, halite, and glauberite) [26]. In dry air, mirabilite dehydrates turning into thenardite (Na_2_SO_4_). Conversely, thenardite can absorb water and turn back into mirabilite. Sulphite salts can impact microbial cells not only by reducing water activity but by increasing ionic strength. For example, studies pertaining to astrobiology have found that sulphate brines are characterised by both low water activity and high ionic strength [27].

For salt to deliquesce, the temperature must be above a critical value and the water vapour near the salt must be above the deliquescence relative humidity, which is a temperature-dependent value. This critical temperature, known as the ‘eutectic temperature’ is the lowest value at which a solution will freeze over all possible mixing ratios. Once the conditions for deliquescence are met, ions would remain in solution until the relative humidity of the air near the brine drops below its deliquescence relative humidity. However, experiments have shown that most salts exhibit a hysteresis effect, recrystallizing (efflorescing) at a much lower value, known as the efflorescence relative humidity. For a concentrated solution of ions (a brine) under equilibrium conditions, the water activity is numerically equivalent to the relative humidity but 100-fold lower (so, for example, 90.7% relative humidity ≡ 0.907 water activity). Deliquescence is best studied for NaCl [28] but also well-studied for other salts including perchlorates [29]. The utilisation of brines formed through deliquescence is a phenomenon observed in the Atacama Desert, where halite (mineralised NaCl) evaporites provide a habitat for microorganisms that take advantage of deliquescence at certain relative humidity levels and temperatures [30,31,32].

In the Venice library, where most of the crystals were observed, *E. halophilicum* was growing near a mean temperature of 22.7 °C (the actual temperatures ranged 22.4 to 23.5 °C; see Supplementary Table S3 in the current manuscript and Table 1 of [33]). In other libraries from which the fungal samples were collected, the mean temperature values ranged between 17 and 22 °C (Supplementary Table S3). Other crystals were observed also in Rome (Library 1) and Genoa where the mean air temperatures in the libraries were 17 °C (with a mean of 42% relative humidity) and 22 °C (with a mean of 61% relative humidity), respectively.

The deliquescence relative humidity for mirabilite at 22.5 °C is 94.7% [34], but the deliquescence properties of eugsterite (and glauberite) are not well known. Thus, the relative humidity near the mirabilite should be higher than 94.7% for a brine to form on the book surfaces. However, supersaturated solutions can form between the deliquescence relative humidities of mirabilite and that of the anhydrous phase, thenardite, i.e., sodium sulphate [34]. At 22.5 °C, thenardite deliquesces at about 83.0% relative humidity [35]; a supersaturated solution with respect to mirabilite could exist between relative humidities of 83.0 and 94.7%. The resulting brine will then remain until the efflorescence relative humidity is reached. For mirabilite at 22.5 °C, dehydration to the anhydrous phase occurs at 78.5% relative humidity. However, a solution (brine) can exist down to the efflorescence relative humidity of thenardite, which can be as low as 55.0% relative humidity at 22.5 °C [35]. Multi-component solutions may exhibit lower deliquescence relative humidity and efflorescence relative humidity values than single-component brines of the constituent salts (e.g., [36,37]). The coexistence of eugsterite and mirabilite may thus broaden the range of conditions where a liquid could persist on the book surfaces.

Although the mean relative humidity in the Venice library was low (i.e., <61%), salt deliquescence on the book surfaces can still occur. All the books on which fungal mycelium grew, independent of the location of the library and the mean values of air temperature and relative humidity, had a water content of ≥8% *w*/*w* (Supplementary Table S3). For paper, a relative humidity of 55.0 to 60.0% is needed at 20 °C for the absorption of enough water vapour to attain a water content of between 8 and 10% *w*/*w* [38]. Furthermore, differences in relative humidity across a room can occur due to air flow, diurnal temperature variations, failure of air conditioning systems, or other factors.

The production of sodium sulphate crystals in the vicinity of *E. halophilicum* colonies indicates that the extracellular milieu has become supersaturated. Hydrophilic polymers, such as extracellular polymeric substances, have a substantial hydration shell and are kosmotropic [39]. In the context of the current study, we believe that this may have acted to trigger the precipitation of sulphate salts. Indeed, it appears that *E. halophilicum* effectively uses brines formed through salt deliquescence as a regulation system. We hypothesise that the *E. halophilicum* was able to regulate biophysical conditions by extracting ions from the book surfaces and dust and (in effect) uses these to obtain water from the atmosphere via salt deliquescence. Furthermore, the dynamics of different salt minerals and different brines appear to produce a supply of bulk water (in the form of briny thin films) and—perhaps of equal importance—these saline phases and phase transitions occur at relative humidities that are equivalent to water-activity values for the most part within the window that the fungus is metabolically active and able to grow (see above). One intriguing question that remains is how the mycelium attracts this concentration of salt in an environment (on books) that is not ion-rich.

Fungi that germinate and grow at ≤0.850 water activity, corresponding to 17% *w/v* NaCl or 50% *w/v* glucose, are defined as xerophilic [40,41]. Some fungi are highly salt tolerant, in some cases able to grow at saturated NaCl (about 35% *w/v* NaCl, 0.755 water activity) (see Figure 5 of [21]). In some cases, halophily is obligate (e.g., *Wallemia ichthyophaga*) because growth occurs well at high NaCl but is poor on glycerol or sucrose [42]. In other cases, fungi are facultatively halophilic, for example *Aspergillus penicillioides* that is both extremely halophilic—see [43]—and can grow at high concentrations of glycerol or sugars, see [21,44]. Tolerance to high salt concentrations is a characteristic of many species within the order Eurotiales and *Eurotium* teleomorphs are abundant in some saline habitats and arid soils. In anthropogenic habitats (within the built environment), *Eurotium* species are biodeteriogens, for example, in dry and salty foods [45]. *Eurotium* is the teleomorphic genus of the *Aspergillus* and *Restricti* sections, most of which have a halophilic and/or xerophilic phenotype [45]. On the surfaces of artifacts, in substrates with a high solute content, or in dust, *E. halophilicum* is thought to act as a pioneer for microbial communities [46].

Stevenson et al. [47] determined the growth kinetics of *E. halophilicum* over a range of biophysical conditions, focusing on the three key parameters known to impact fungal metabolism: water activity, temperature, and pH [48,49]. The culture media were supplemented with glycerol + NaCl + sucrose to reduce water activity (to between 0.995 and 0.651) and adjusted by addition of buffers (citric acid/Na^2^PO_4_, PIPES/NaOH, or HEPES/NaOH) to modify pH (2.80 to 9.50). Incubations were carried out at temperatures from 2 to 50 °C. *Eurotium halophilicum* exhibited water-activity windows for germination and growth that spanned from ≥0.961 to 0.651, with a maximum rate of spore germination at around 0.900 water activity and no germination at 0.995 water activity [47]. The fungus could also germinate readily over the entire pH range tested, and had an optimum germination temperature of 30 °C, which is typical of other extremely xerophilic fungi [47,50]. We strongly suspect that *E. halophilicum* uses a multi-faceted strategy to attain these levels of stress tolerance (including production of microfilaments, compatible solutes, and extracellular polymeric substances, and the use of ion/salts to obtain vapour-phase water and regulate water activity in the form of brines). There is also evidence that fungi in the *Restricti* section are highly efficient at energy generation, thus supporting their stress biology [51,52].

We believe that the hair-like microfilaments extending from the *E. halophilicum* act to absorb water vapour and collectively act to retain a layer of humid air close to the hyphal surface. Similar microfilaments have been reported along the stipes of the conidiophores for other fungi in the *Restricti* section of the *Aspergillus* genus [53]. Such microfilaments have been used as a morphological characteristic of individual xerophiles [53,54]: the conidiophores of *Aspergillus restrictus* appear densely covered with microfilaments, *Aspergillus salinicola* microfilaments are less dense, and microfilaments are absent in *Aspergillus vitricola,* which has smooth hyphae. The *E. halophilicum* microfilaments are longer than those of *A. restrictus* and the former appear to be sticky, as they sometimes extend from one hypha to another (Figure 2a). The composition of these microfilaments has yet to be determined, but they likely consist of cell wall material [55].

Fungi, including *Eurotium* species, synthesise and accumulate low molecular weight organic substances that are compatible with metabolism and can be used for osmotic adjustment (known as compatible solutes) such as glycerol and arabitol [17,56,57]. According to Zajc et al. [58], halophilic fungi maintain low concentrations of intracellular ions (such as Na^+^) and use glycerol and other polyols for osmotic adjustment, as do xerophilic fungi that are not halophiles [59,60]. Other strategies to mitigate osmotic stress and desiccation include the production of extracellular polymeric substances [61,62]. Extracellular glycoproteins are produced by many fungi growing in soils containing high salt concentrations [63] and, as kosmotropes, can bind water thus acting to maintain water in the vicinity of the cells [64]. If low water availability is caused by the absence of water (rather than the presence of osmolytes in the extracellular environment) [65], the fungus must absorb water from the vapour phase if it is to maintain sufficient cell turgor for germination and growth.

*Eurotium halophilicum* produces abundant extracellular polymeric substances and predominantly concentrates Na, S, and Ca and P and K in conidia. In the published literature, there is a paucity of information on the uptake of mineral nutrients by *E. halophilicum* mycelium when the fungus is cultured in vitro on a nutrient medium containing salts. In the current study, however, we found that this fungus was able to concentrate some chemical elements inside the cells and around its mycelium (in the extracellular polymeric substances), possibly extracting them from the surfaces of the books. The book covers from which *E. halophilicum* were made of a range of materials such as parchment, leather, cardboard, hemp, linen, cotton, and/or buckram cloth (polyester fabric coated in acrylic coating). The presence of chemical elements in these materials can be highly variable, both in type and concentration. Furthermore, chemical elements such as Na, S, Cl, K, and Ca may be contained in the dust that settles on books [66,67].

It was not possible to analyse the composition of the covers where the mycelia developed because this would have been destructive and so incompatible with the conservation policy. However, the results of the current study suggest that *E. halophilicum* could actively concentrate, assimilate, and compartmentalise Na, S, and Ca into the extracellular polymeric substances, and P and K in their conidia (Figure 5). The strong correlation between P and K (Table 4), is consistent with the findings from a previous study, where a positive correlation was observed between cellular levels of P and K, especially in ectomycorrhizal fungi [68]. Potassium has also been shown to be one of the main counterions of polyphosphate granules, mainly located in fungal vacuoles [69]. The uptake of P and the synthesis and accumulation of polyphosphates in vacuoles is a mechanism used by fungi to activate cation import (the negative charge of polyphosphate is balanced by the uptake of cations, like K and sometimes Na) to maintain overall cellular charge neutrality [70].

There are currently no data on the elemental composition of *E. halophilicum* mycelium that developed on book surfaces made of different materials. The salts that *E. halophilicum* concentrates in its mycelium may originate from the materials themselves [71,72] (and possibly also from the dust deposited on them). The micronutrients (nutrients other than C or N) present in paper and parchment can vary greatly depending on the manufacturing process [71,72,73]. Furthermore, these substances (Na, P, S, K, etc) can be uniformly distributed within these materials (especially if they were used during their manufacture) or heterogeneous/patchy in distribution (for example, if they originate in dust) [74]. Whether or not the fungus takes in all types of ions from the substrate is uncertain, but we do suspect that the production of extracellular polymeric substances can effectively concentrate ions. That fungi can transport certain elements over long distances through the mycelium is well known, especially from studies of mycorrhizal fungi [75], or the decay of wood, glues and straw used in construction and degradation of mortar, plaster, and other building materials [76]. For C, N and Pthese fluxes from areas of high concentration to nutrient-poor parts of the mycelium have been measured using isotopes [77]. In the case of cations such as Na, K, Ca, or metals there are not many studies, although in soil and especially in decaying leaf litter, fungi are known to be involved in the biogeochemical cycles of these elements and can actively translocate and concentrate them [78]. Boswell et al. [79] demonstrated that translocation between areas of different nutrient availability in the mycelium allows fungi to colonise substrates with low initial resource availability. Therefore, the translocation of nutrients by fungi is a response to environmental heterogeneity and can itself generate heterogeneity within the habitat.

According to Polo et al. [46], the hygroscopicity of the external surfaces of the books (e.g., spine and the edges of the pages) together with fluctuating thermo-hygrometric conditions seem to favour the (stress tolerant) species *E. halophilicum*. Consistent with this, the spread of *E. halophilicum* in libraries has been correlated with poor ventilation, such as that of densely arranged Compactus^®®^ shelving [10,11,12]. Small temperature fluctuations in the still air close to books can cause a higher relative humidity that favours water condensation on surfaces when the air temperature reaches the dew point. This creates instant but ephemeral niches where some fungal conidia can germinate (we assume that the presence of ions is also needed for the germination of *E. halophilicum*; see [47]). For example, the drop in temperature from the range 20 to 24 °C to values around 15 to 18 °C, when the relative humidity of the air is maintained at 65%, can lead to water condensation on book covers. A relative humidity of 65% is considered suitable for conservation by some institutions that archive books. However, several fungi are known to proliferate below this relative humidity (that is equivalent to 0.650 water activity) [22]; for *A. penicillioides* even down to 0.585 water activity (equivalent to 58.5% relative humidity) [43]. Furthermore, extreme xerophiles (including *A. penicillioides*) are known to cause foxing of books and fabrics [80].

## 4. Materials and Methods

### 4.1. Sampling Procedure

Mycelium samples were obtained by pressing a carbon-based, impurity-free adhesive tape (FungiTape^TM^, Scientific Device Lab., Inc., Glenview, IL, USA) onto the fungal colonies found on the spines of books in five Italian libraries from Genoa, Rome (two libraries), Turin, and Venice [12,33]. This way, it was possible to collect the hyphae and reproductive structures (mainly conidiophores and conidia) whilst retaining their relative positions. More than 200 books were sampled, with two or three fungal colonies collected from each book. However, the number of books analysed differed in each library, with 29 books sampled in Genoa, a total of 73 books sampled in Rome (67 from Library 1 and six from Library 2), and 13 books sampled in Turin, and 90 books sampled in Venice. During the 6-h sampling procedures, the indoor temperature and air relative humidity in Venice’s library were monitored by means of a portable thermo-hygrometer data logger HD2101.1 (Delta OHM, Padua, Italy; temperature range: −20 to +65 °C, resolution: 0.1 °C, accuracy: ±0.1 °C; relative humidity range: 0 to 100%, resolution: 0.1% relative humidity, accuracy: ±0.1%), recording the data every 5 min. The monitoring was performed in the middle of the library, 1 m above the floor, at the location where the sampling of books took place. In the libraries or archival repositories from Genoa, Rome and Turin, air temperature and relative humidity were measured with hygrolog-type detection sensors (Rotronic^®^ HygroClip, Process Sensing Technologies PST Srl, Rho, Italy) capable of recording humidity and temperature values with accuracy: +/−0.1%. The mean values were derived from measurements taken across a minimum period of one month (the time-period varied according to the libraries). Despite the use of different models of thermo-hygrometers to record environmental values of temperature and relative humidity in the different libraries, the data were obtained with comparable resolution and accuracy and in each case, the instruments were calibrated in accordance with the manufacturer’s instructions. Although the measurements were taken over a range of time spans, they always coincided with a situation of visible contamination of the books. The water content of the books showing mould growth on their cover was also measured by means of a paper hygrometer (Aqua-Boy PMII—Paper, Cardboard Moisture Meter, 4 to 12% w/w scale, Aqua-Boy, UK, Accuracy: ±0.1. The identification of the fungus was based on microscopic morphology, ability to grow on specific substrates [4,5,7] and molecular markers [81]. The isolation of the fungus was performed previously by placing the adhesive tapes onto Malt Extract Agar with 15% *w/v* NaCl (MEA 15%) or Dichloran Glycerol Agar (DG18, containing 18% *w/v* glycerol) [12,33]. The strains were deposited at the Mycotheca Universitatis Taurinensis (MUT), Department of Life Sciences and System Biology, University of Turin (Italy) with the following codices: MUT 1313-16, MUT 1298, MUT 1303, and MUT 1322.

### 4.2. Scanning Electron Microscopy

The adhesive tape fragments used to retrieve the fungal structures were cut and fixed onto 12-mm diameter metal stubs using double-sided carbon tape. The adhesive tape samples were observed with a Zeiss EVO50 scanning electron microscope (Carl-Zeiss Electron Microscopy Group, Oxford, UK). The samples were observed under variable pressure without pre-treatment at approximately 50 Pa, at which most of the fungal structures did not change shape, and associated compounds did not evaporate [82]. A tungsten filament fed the electron beam of the EVO50. Both a backscattered electrons detector and a secondary electrons detector were used. Some samples were instead gold-coated without a previous fixation procedure because the fungal structures appeared naturally dried, and rehydration would have introduced artifacts [82]. This metallisation was conducted with a Baltec Sputter Coater and samples were observed using SEM in high vacuum mode [83]. Sputtering was performed under a flow of argon gas at a working distance of 50 mm at 0.05 mbar and a current of 40 mA for 60 s to create a gold film approximately 15-nm thick. Both metallised and non-metallised samples were analysed.

### 4.3. X-ray Powder Diffraction

X-ray powder diffraction spectra were recorded with a Philips PW1050 powder diffractometer (based on Bragg–Brentano parafocusing geometry). A nickel-filtered Cu Kα1 radiation (λ = 0.15406 nm) and a step-by-step technique (step of 0.05° in 2θ and the acquisition spectra were 5° to 100° 2θ) with collection times of 10 s/step were employed. The X-ray diffraction patterns were collected and processed by Crystallographica Search-Match (CSM—Version 3.1.0. 0 © 1996–2008, Oxford Cryosystems, Oxford, UK). These analyses of *E. halophilicum* and salt crystals were performed directly on the samples on the adhesive tapes.

### 4.4. Energy-Dispersive X-ray Spectroscopy

Energy-dispersive X-ray spectroscopy was performed with an INCA Oxford 250 system, maintaining the electron beam at 20 keV, with a mean working distance to the sample of 12.5 mm. The calibration of the apparatus was based on the standards CaCO_3_, SiO_2_, albite, MgO, Al_2_O_3_, GaP, FeS_2_, wollastonite, feldspar MAD-10, Ti and Fe, supplied by Agar Scientific Ltd. (Stansted, UK) and the conventional ZAF correction (atomic number Z, absorption A, fluorescence F) from the Oxford INCA 250 software was applied to the measurements to convert apparent concentrations (raw peak intensity) into (semi-quantitative) concentrations corrected for inter-element matrix effects [83]. A total of 205 energy-dispersive X-ray spectra acquired in variable vacuum mode, on non-metallised samples, were used for statistical analysis. Moreover, 23 spectra obtained from crystals that were analysed in high-vacuum mode were used to calculate stoichiometric ratios between elements. The data analysed were derived from samples taken in all five libraries (for the raw data, see the link provided in the Data Availability Statement).

### 4.5. Statistical Analysis of Energy-Dispersive X-ray Spectroscopy Data

Energy-dispersive X-ray spectroscopy measurements were repeated on a representative number of structures for each sample analysed. The resulting dataset was processed using one-way analysis of variance (ANOVA) with 95% confidence to assess the relationships between variables (weight per cent of chemical elements) and the significance of differences between different structures (e.g., conidia and adhesive-tape background) within each sample. An ‘unbalanced’ ANOVA model was applied as the number of observations within each category differed. The ANOVA analysis was followed by Tukey’s post hoc test. Principal component analysis was then used to visualise the correlations between the chemical elements [84] and reduce the number of variables for further statistical analysis. The factor scores obtained for the first three principal components resulting from the analysis were used to perform discriminant analysis, which requires 3 to 20 times as many observations as variables [84]. The linear dependence (correlation) between the chemical elements in the energy-dispersive X-ray spectroscopy dataset was measured using Pearson’s coefficient ‘R’. The resulting coefficient has a value that ranges between +1 and −1, with +1 indicating complete positive linear correlation, 0 no correlation, and −1 complete negative linear correlation. The *p*-values computed for each coefficient tested a null hypothesis that the coefficients were not significantly different from 0 (with a significance level of 0.05%) [85]. The ANOVA, principal component analysis and discriminant analysis analyses were performed with XLSTAT 2019.3.2 software (Addinsoft, Paris, France).

The electron beam can be focused on very small areas, in the order of nm^2^, allowing a surface resolution that readily resolves objects that are a few tens of nm in dimension. This way, a database was obtained with repeated observations of the composition of conidia and other fungus structures carried out on samples from the covers of different books (see the link provided in the Data Availability Statement). Some samples were analysed with energy-dispersive X-ray spectroscopy also after metallisation in order to be able to focus on peculiar structures like the crystals (Figure 3c). When this was the case, the spectra obtained contained gold, also present in the background (Supplementary Figure S1).

## 5. Conclusions

We believe that *E. halophilicum* uses salt deliquescence as an integral part of its water-relations strategy. Further studies are needed to confirm that the kosmotropicity of extracellular polymeric substances contributes to the concentration of ions, which in turn impacts the precipitation of the salts. Such studies will give insights into the possibility that salt deliquescence is in this way influenced by the fungus albeit that the deliquescence event is still driven by (abiotic) physical chemistry. Whereas *E. halophilicum* is not considered the most-halophilic fungus, it has the remarkable ability to grow at water activities lower than that of saturated NaCl and may have value as a model system for use in astrobiology-related studies [86]. We believe that *E. halophilicum* combines the creation of a large surface area capable of absorbing moisture by the production of fine microfilaments on the hyphae (that can also trap a layer of humid air), secretion of abundant extracellular polymeric substances, production of compatible solutes, transport, assimilation, and concentration of elements from the substrates to the mycelium, and the use of salt deliquescence as a coordinated and effective water-regulation strategy. Collectively, these adaptations appear to allow the fungus to maintain hydration in both space and time.

It should be noted that even during desiccation, fungal cells are dependent on water relations because their residual water preserves macromolecular structures and the mechanics, kinetics, and cellular biology of the rehydration process are critical to survival (see [87] and references therein). For xerophilic fungi that live on surfaces, the ability to persist in an anhydrobiotic condition and to survive sudden rehydration are essential phenotypic traits. Their ability to cope with both instantaneous and long-term changes in conditions is dependent on the versatility of their stress biology. *Eurotium* is commonplace in dried foods and feeds [4,5,8] that are known to spoil due to fungal growth even under climate-controlled conditions. We believe that the use of salt deliquescence by *E. halophilicum* as a way to access liquid water is not unique to book surfaces, and that food-spoilage events that are not currently understood may be in fact caused by fungal proliferation within thin brine films invisible to the naked eye.

Global warming is having a profound effect on the behaviour and distribution ranges of microfungi, macrofungi, and invertebrates and other organisms (e.g., [88,89,90]). This trend is also seen for halotolerant and halophilic microbes in marine and other saline ecosystems that are currently undergoing traumatic change [91,92]. Indeed, if left unchecked, global warming may ultimately impair the habitability of Earth’s surface [93]. The ecology of *E. halophilicum* is complex in as much as—like other fungal xerophiles—this species is commonly found on dry surfaces in nature and surfaces of artifacts and dust particles [50,65,94,95,96]. Global climate change is causing various perturbations at the regional scale, at some places/times causing drought and at others causing floods. Similarly, at the scale of the surface habitats of microbes, water availability may in some cases increase and in other cases decrease. Within the built environment, we do not expect major climate change-induced changes in the ecology of *E. halophilicum* where there is the regulation of temperature and relative humidity. Nevertheless, there is some evidence of climate change impacts even under climate-controlled conditions [15]. Furthermore, there are libraries, museums, art galleries, and other archives located in many regions of the world which lack air-conditioning systems. In such cases, changes in local climatic conditions will profoundly impact the ecology of fungi on surfaces. Global warming is increasing the water retention by Earth’s atmosphere [1,2], so there will likely be on average an increase in the frequency of water being available to such fungi growing on surfaces, whether or not this is obtained by deliquescence. We believe that the current episode of global warming will favour the prevalence of *E. halophilicum* and that of other fungal xerophiles commonly found on surfaces in natural ecosystems.

## Figures and Tables

**Table 1:** Mean values (*n* ≥ 30) of elemental composition as weight percentage (*w*/*w* %) of conidia, extracellular polymeric substances, and background (adhesive tape). Values in each column labelled with a different letter (a, b, or c) are significantly different for *p* < 0.001 based on one-way ANOVA and Tukey’s post hoc test. Pr = probability from the ANOVA; F = the ratio of the variances from the ANOVA. The data analysed were derived from samples taken in all five libraries (for the raw data, see the link provided in the Data Availability Statement).

Categories of Observations	C	O	Na	P	S	Cl	K	Ca
Conidia	76.250 b	21.954 b	0.175 b	0.233 a	0.819 b	0.173 a	0.361 a	0.036 b
Extracellular polymeric substances	65.861 c	25.654 a	3.617 a	0.013 b	3.054 a	0.145 a	0.133 b	1.524 a
Background	92.379 a	7.591 c	0.000 c	0.000 c	0.031 c	0.000 b	0.000 c	0.000 c
Pr > F	<0.0001	<0.0001	<0.0001	<0.0001	<0.0001	0.0001	<0.0001	<0.0001
Significant	Yes	Yes	Yes	Yes	Yes	Yes	Yes	Yes

**Table 2:** Eigenvalues and variability of the first three orthogonal principal components used in the discriminant analysis (i.e., F1, F2, and F3). The principal component analysis was used to reduce the variability of the dataset consisting of repeated energy-dispersive X-ray spectroscopy observations, with the chemical elements as variables and the observed objects (conidia, extracellular polymeric substances, and background) as observations.

Parameters of the Principal Components	F1	F2	F3
Eigenvalue	3.615	2.072	1.048
Variability (%)	45.186	25.898	13.095
Cumulative variability (%)	45.186	71.084	84.179

**Table 3:** Discriminant analysis classification, showing the correctness of *a priori* knowledge of group membership of each object (conidia, extracellular polymeric substances, and background [adhesive tape]) based on their elemental composition.

From\To	Conidia	Extracellular Polymeric Substances	Background	Total	Correct (%)
Conidia	68	11	3	82	82.93
Extracellular polymeric substances	16	67	10	93	72.04
Background	5	1	32	38	84.21
Total	89	79	45	213	78.40

**Table 4:** Correlation matrix showing Pearson’s coefficients ‘R’ for each pair of chemical elements are shown above the grey shading, with +1 indicating a perfect positive linear correlation, 0 no correlation, and −1 a perfect inverse linear correlation. The *p*-values (below the grey shading) computed for each coefficient indicate the statistical significance of the correlation (*p* < 0.05). The data analysed were derived from samples taken in all five libraries (for the raw data, see the link provided in the Data Availability Statement).

Variable	C	O	Na	P	S	Cl	K	Ca
C		−0.92	−0.77	−0.20	−0.71	0.04	−0.18	−0.63
O	<0.0001		0.56	0.13	0.45	0.03	0.12	0.44
Na	<0.0001	<0.0001		−0.12	0.82	−0.12	−0.13	0.61
P	0.003	0.05	0.08		−0.07	−0.07	0.97	−0.06
S	<0.0001	<0.0001	<0.0001	0.34		−0.08	−0.09	0.54
Cl	0.59	0.66	0.08	0.31	0.22		−0.06	−0.15
K	0.01	0.07	0.06	<0.0001	0.18	0.36		−0.07
Ca	<0.0001	<0.0001	<0.0001	0.41	<0.0001	0.02	0.29	

## Data Availability

The datasets generated during the current study are available from https://zenodo.org/deposit/7383183, accessed on 15 September 2022.

## Supplementary Materials

The following supporting information can be downloaded at: https://www.mdpi.com/article/10.3390/pathogens11121462/s1, Figure S1: *E. halophilicum* mycelium sampled directly from books (that had been kept at about 20 °C) and observed with a scanning electron microscope (SEM EVO50 Zeiss, Oxford) in high vacuum mode, after metallisation with gold; Images (**a**,**b**) show how data were acquired by energy-dispersive X-ray spectroscopy and how different fungal structures were classified *a priori*. Locations within samples where acquisitions were made (conidia, crystals, and background adhesive tape) (indicated by arrows in (**a**,**b**); Figure S2: Principal components analysis of the elements analysed by energy-dispersive X-ray spectroscopy from different fungal structures: conidia, extracellular polymeric substances (EPS), background adhesive type: (**a**) correlation circle plot obtained on the first two coordinates F1 and F2, and (**b**) biplot with the projection of all the variables (chemical elements) on the plane generated by factors F1 and F2; Figure S3. Typical X-ray diffraction patterns and mineral phases from the adhesive tape containing the *E. halophilicum* with the sodium sulphate crystals; Tables S1 and S2: List of X-ray diffraction peaks and peaks identifying eugsterite and mirabilite crystals; Table S3: Water content of books, air temperature, and relative humidity in libraries.
