# Supplementary material for: Biophysical Manipulation of the Extracellular Environment by Eurotium halophilicum
Source: Pathogens. 2022 Dec 2;11(12):1462. doi: 10.3390/pathogens11121462 (PMC9781259; doi:10.3390/pathogens11121462)
Supplement: Supplementary file 1 [file pathogens-11-01462-s001.zip › pathogens-2068113-supplementary.pdf]

pathogens

MDPI

Supplementary material

Biophysical manipulation of the extracellular environment by *Eurotium halophilicum*

Anna Micheluz [1], Flavia Pinzari [2,3,*], Edgard G. Rivera-Valentín[4], Sabrina Manente[5], and John E. Hallsworth[6]

1. Conservation Science Department, Deutsches Museum, Museumsinsel 1, 80538 Munich, Germany
2. Institute for Biological Systems, Council of National Research of Italy, Area della Ricerca di Roma 1, Via Salaria Km 29,300, 00015 Monterotondo (RM), Italy
3. Life Sciences Department, Natural History Museum, Cromwell Road, London SW7 5BD, UK
4. Applied Physics Laboratory, Johns Hopkins University, Laurel, MD 20723, USA
5. Department of Molecular Sciences and Nanosystems, Scientific Campus, Ca' Foscari University of Venice, via Torino, 30170 Venice, Italy
6. Institute for Global Food Security, School of Biological Sciences, Queen's University Belfast, 19 Chlorine Gardens, Belfast BT9 5DL, UK

* Correspondence: flavia.pinzari@cnr.it or f.pinzari@nhm.ac.uk

---

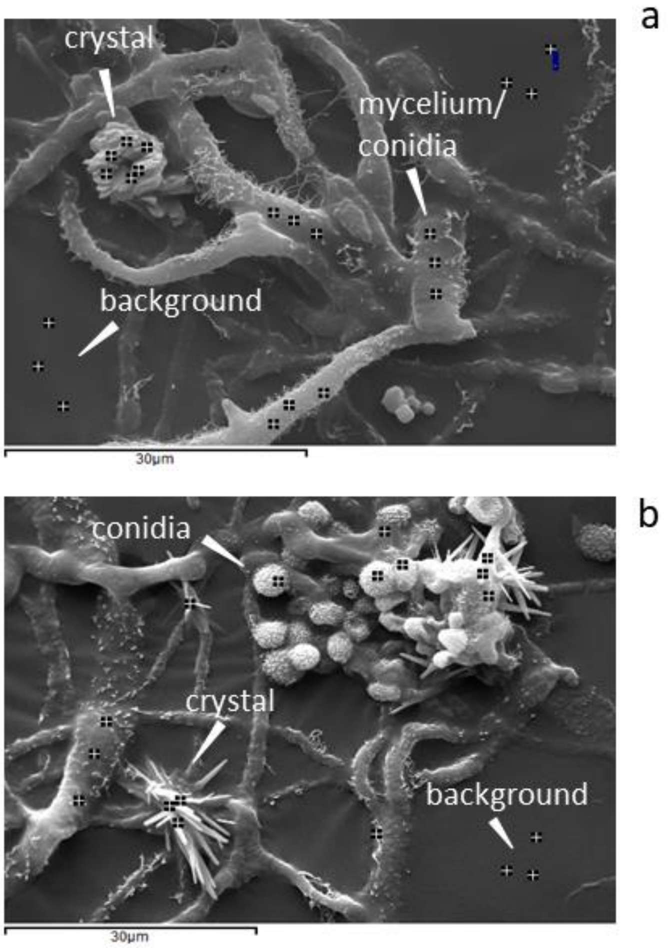

Supplementary Figure S1. *Eurotium halophilicum* mycelium taken from books (that had been kept at a mean temperature of 22.7±0.5 °C) and observed with a scanning electron microscope (SEM EVO50 Zeiss, Oxford) in high vacuum mode, after metallisation with gold; Images **(a)** and **(b)** show how data were acquired by energy-dispersive X-ray spectroscopy and how different fungal structures were classified *a priori*. Locations within samples where acquisitions were made (conidia, crystals, and background) (indicated by arrows in **a** and **b**).

pathogens

MDPI

Supplementary Figure S2. Principal components analysis of the elements analysed by energy-dispersive X-ray spectroscopy from different fungal structures: conidia, extracellular polymeric substances (EPS), and background: **a**) correlation circle plot obtained on the first two coordinates F1 and F2, and **b**) biplot with the projection of all the variables (chemical elements) on the plane generated by factors F1 and F2.

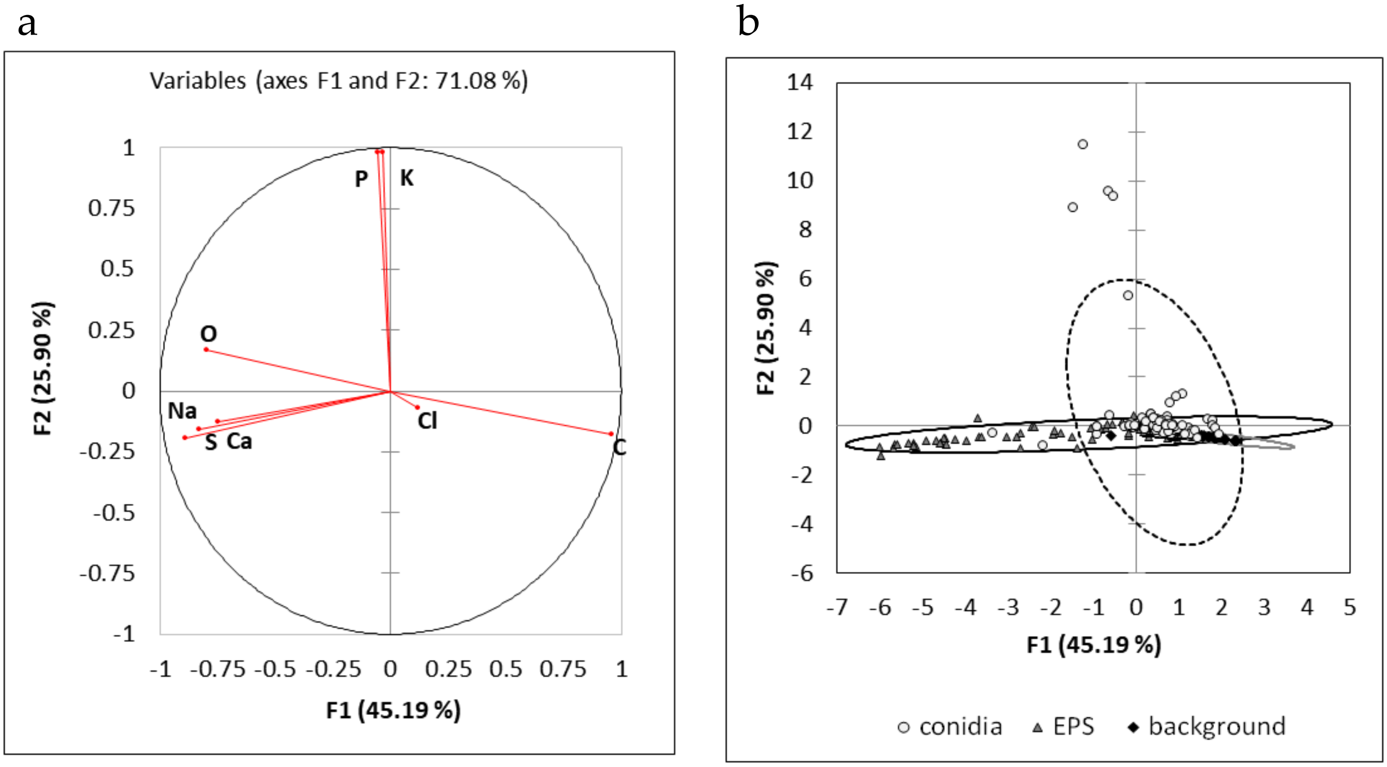

pathogens

MDPI

Supplementary Figure S3. X-ray powder diffraction spectra with mineral phases from the adhesive tape containing the *Eurotium halophilicum* and the sodium sulphate crystals. The spectra were recorded with a Philips PW1050 powder diffractometer (Bragg–Brentano parafocusing geometry). A nickel-filtered Cu Kα1 radiation (λ = 0.15406 nm) was used with a step-by-step technique (step of 0.05° in 2θ and the acquisition spectra are 5° to 100° 2θ). Collection times of 10 s/step were employed. The XRD patterns were collected and processed by Crystallographica Search-Match (CSM - Version 3.1.0. 0 © 1996-2008, Oxford Cryosystems).

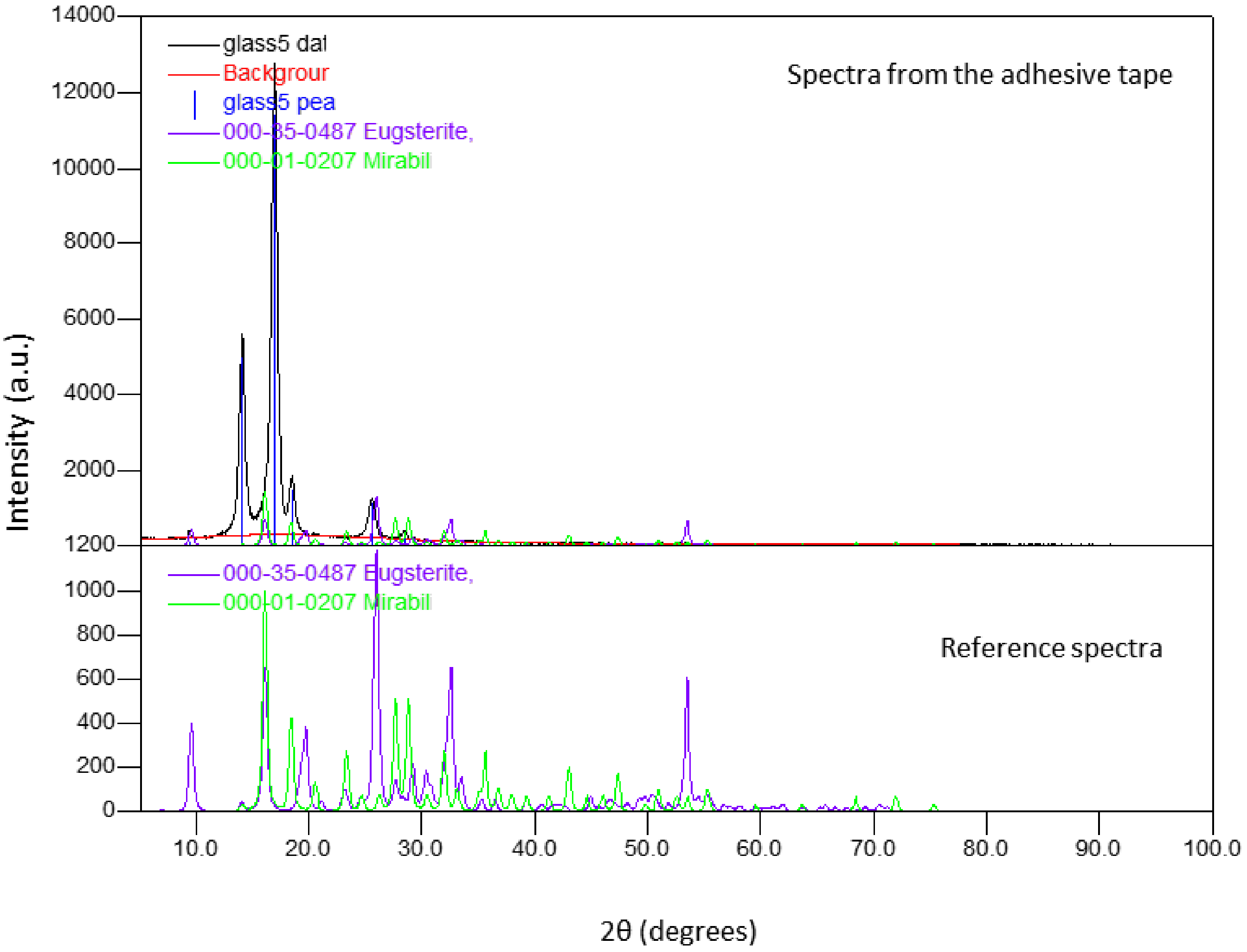

pathogens

MDPI

Supplementary Table S1. Summary of the results obtained analysing the adhesive tape containing the *Eurotium halophilicum* and the sodium sulphate crystals with X-ray powder diffraction. The observed spectra showed peaks corresponding to the minerals eugsterite and mirabilite. However, only some of the peaks could be matched with reference spectra contained in the Crystallographica Search-Match (CSM - Version 3.1.0. 0 © 1996-2008, Oxford Cryosystems) database. The figures of merit are a measure of the quality of a given phase set.

	**Eugsterite (A)**	**Mirabilite (B)**
Formula	$Na_4 Ca (SO_4)_3 \cdot 2 H_2 O$	$Na_2SO_4 \cdot 10 H_2 O$
Figure of Merit	5%	7%
Total Peaks	93	32
Peaks Matched	14	6
New Matches	14	6
Strong Unmatched	1	0
Peak Shift	0	0
Scale Factor	0.0889365	0.116253

Supplementary Table S2. List of peaks obtained analysing the adhesive tape containing the *Eurotium halophilicum* and the sodium sulphate crystals with X-ray powder diffraction. The matches are peaks corresponding to the minerals eugsterite (A) and mirabilite (B). The data processing and search for the similarity of peaks with the spectra of known minerals was performed with the software Crystallographica Search-Match (CSM - Version 3.1.0. 0 © 1996-2008, Oxford Cryosystems).

Confidence Threshold: 90%
Matched / Total: 2 / 7

2-Theta	*D-Spacing*	*Intensity*	*Width*	*Confidence*	*Matches*
9.408	9.3925	168	0.351	100%	
14.038	6.3035	5004	0.357	100%	AB
16.913	5.2380	11433	0.360	100%	
18.551	4.7790	1526	0.517	100%	B
25.566	3.4814	1182	0.401	100%	
28.417	3.1382	235	0.395	100%	
76.024	1.2508	18	0.315	92.5%	

pathogens

MDPI

Supplementary Table S3. Water content (%) of the books where *Eurotium halophilicum* samples were collected. The measures were obtained using a paper hygrometer. Average indoor temperature (°C) and air relative humidity (%) were measured in the libraries. The values are the average of a minimum of three weeks of monitoring performed with portable or permanent thermo-hygrometers.

	**Mean water content of books (%)**	**Air relative humidity (%)**	**Air temperature (°C)**	**Presence of crystals**
Rome (Library 1)	8±0.3	43±8.5	17±1.6	Yes
Genoa	9±0.2	61±3.2	22±2.1	Yes
Venice	10±1.1	54.7±0.7	22.7±0.2	Yes
Turin	8±0.1	55±4.0	20±1.0	No
Rome (Library 2)	9±0.2	63±2.0	18±1.1	No
